# The association between the triglyceride-glucose index and in-stent restenosis in patients undergoing percutaneous coronary intervention: a systematic review and meta-analysis

**DOI:** 10.1186/s12872-024-03903-1

**Published:** 2024-05-03

**Authors:** Haodong Jiang, Yuntong liu, Haoyu Guo, Zhihao Liu, Zhibo Li

**Affiliations:** 1 Department of Cardiovascular, The Second Hospital of Jilin University, Changchun, China; 2 Department of Endocrinology, Dalian Central Hospital, Dalian, Liaoning China

**Keywords:** Triglyceride-glucose index, In-stent restenosis, Meta-analysis, Insulin resistance, Coronary atherosclerotic heart disease

## Abstract

**Background:**

Insulin resistance (IR) can lead to cellular metabolic disorders, activation of oxidative stress, and endothelial dysfunction, contributing to in-stent restenosis (ISR). The triglyceride-glucose index (TyG index), a new indicator reflecting IR, is extensively researched in the cardiovascular field. This study, through a meta-analysis, aimed to utilize a larger combined sample size and thereby enhance the overall test efficacy to explore the TyG index-ISR relationship.

**Methods:**

A thorough search was conducted in the PubMed, EMBASE, Web of Science, and Cochrane Library databases to find original papers and their references published between 1990 and January 2024. This search included both prospective and retrospective studies detailing the correlation between the TyG index and ISR in individuals with coronary heart disease (CHD).

**Outcomes:**

The five included articles comprised 3,912 participants, and the odds ratio (OR) extracted from each study was combined using the Inverse Variance method. Results showed that, in the context of CHD patients, each incremental unit in the TyG index, when treated as a continuous variable, corresponded to a 42% elevation in ISR risk (95% CI 1.26–1.59, I²=13%, *p* < 0.005). When analyzing the TyG index categorically, the results revealed a higher ISR risk in the highest TyG index group compared to the lowest group (OR: 1.69, 95% CI 1.32–2.17, I²=0). Additionally, in patients with chronic coronary syndrome (CCS), each unit increase in the TyG index, the risk of ISR in patients increased by 37% (95% CI 1.19–1.57, I²=0%, *p* < 0.005). This correlation was also observable in acute coronary syndrome (ACS) patients (OR:1.48, 95% CI 1.19–1.85, I²=0, *p* < 0.005).

**Conclusions:**

The TyG index, an economical and precise surrogate for IR, is significantly linked with ISR. Furthermore, this correlation is unaffected by the type of coronary heart disease.

**Supplementary Information:**

The online version contains supplementary material available at 10.1186/s12872-024-03903-1.

## Background

The advent of percutaneous coronary intervention (PCI) has markedly reduced coronary atherosclerotic heart disease (CHD) mortality rates in recent years [1, 2]. However, stent implantation in the coronary artery can cause vascular injury and neointimal hyperplasia, leading to the occurrence of in-stent restenosis (ISR). According to reports, the occurrence of ISR in the new generation of drug-eluting stents is still about 3-10% [3, 4], and approximately 25% of patients with ISR present with acute myocardial infarction [5]. Although the incidence of ISR is low, considering the large number of people undergoing stent implantation for revascularization and the poor prognosis of ISR, it is crucial to discover groups at high risk for ISR.

Insulin resistance(IR) refers to an experimental or clinical phenomenon characterized by impaired insulin function in stimulating glucose uptake by tissue cells. This condition can lead to cellular metabolic disorders, activation of oxidative stress response, and endothelial dysfunction, making it a risk factor for ISR [6]. Recently, numerous studies have confirmed a close link between the triglyceride-glucose index (TyG) and IR. Unlike the time-consuming and expensive hyperinsulinemic-euglycemic clamp technique (HEC), the TyG offers a simpler approach to evaluating IR. It does not require insulin measurements. Instead, it relies on blood glucose and lipid measurements, making it suitable for widespread use in clinical practice [7]. A meta-analysis of 25,656 participants revealed that individuals with a high TyG index faced increased risks of CHD and had poor outcomes [8]. Observational studies also suggested a higher propensity for ISR in CHD patients with elevated TyG index levels. However, some of these studies have limitations due to their small sample sizes [9, 10]. Therefore, this meta-analysis aimed to overcome these limitations by combining sample sizes and thereby enhancing the overall test efficacy to explore the TyG index-ISR relationship.

## Method

The composition of this meta-analysis adhered to the Preferred Reporting Items for Systematic Reviews and Meta-analyses (PRISMA) statement [11]. Furthermore, it was registered on the Prospero website (PROSPERO (york.ac.uk)) under registration number CRD42023482588. In the PubMed, EMBASE, Web of Science, and Cochrane Library databases, literature published between 1990 and 2024 was independently searched by two authors (H.J and Y.L). The search utilized the following keywords: ‘stent,’ ‘stents,’ ‘stenting,’ ‘fasting glucose and triglyceride,’ ‘fasting glucose and triglycerides,’ ‘fasting plasma glucose and triglycerides,’ ‘fasting triglyceride-glucose index,’ ‘fasting plasma glucose and triglycerides index,’ ‘fasting triglycerides-glucose index,’ ‘glucose and triglycerides,’ ‘glucose and triglycerides index,’ ‘glucose-triglyceride index,’ ‘triglyceride and glucose index,’ ‘triglyceride x glucose index,’ ‘triglyceride-glucose index,’ ‘triglycerides x glucose,’ ‘triglycerides-glucose index,’ ‘TyG index,’ and ‘triglyceride-glucose index’. In cases of discrepancies during the search, the third author (Z. L) was consulted. Furthermore, we also meticulously screened the references of original and review articles. The search process did not restrict language. (Details can be found in Additional file 1).

### Inclusion and exclusion criteria

Eligibility for studies based on these criteria: (1) the research was either prospective or retrospective in design; (2) the study focused on the TyG index in relation to coronary in-stent restenosis; (3) the outcomes were reported as odds ratio (OR), relative risk (RR), or hazard ratio (HR) with corresponding 95% confidence intervals (CIs). Exclusion of studies was based on these criteria: (1) non-human subjects; (2) studies lacking a control group; (3) research with inaccessible or unextractable data; (4) non-original research publications, including editorials, reviews, and letters to the editor.

### Data extraction and quality assessment

The relevant data were extracted by two authors (H.J and Y.L). In case of disagreements, consultation with a third author (Z.L) was sought. Additionally, they separately recorded the following details: primary author, publication date, study design, duration of follow-up, geographical location of the study, sample size, demographic characteristics of patients, type of TyG index variable used, variables adjusted in multivariate regression analysis, and effect size. The quality assessment of the included studies was performed using the Newcastle-Ottawa Scale (NOS), which allocates a score between 0 and 9 [12]. Studies that scored six or higher were categorized as possessing high methodological quality.

### Statistical analysis

Given that ISR is a relatively low-probability event, it is commonly assumed that HR, RR, and OR are approximately equal (as in most studies) [13]. The OR extracted from each study was combined using the Inverse Variance method [14]. When analyzing the TyG index categorically, we extracted OR for ISR by comparing patients in the highest TyG index group to those in the lowest TyG index group. In analyzing the TyG index continuously, we extracted the OR for ISR with per unit increment in the TyG index.

The Cochran Q test and I^2^ statistic were used to assess heterogeneity, which was considered significant if I² exceeded 50% or the p-value was less than 0.05. Considering that we included only five studies and there were significant differences in study sizes among them, we opted for a fixed-effects model [15, 16]. The forest plot presented the combined results of the studies. To evaluate potential publication bias, we utilized funnel plots. Additionally, when necessary, Egger’s test and the trim-and-fill method were applied to further evaluate publication bias [17]. Sensitivity analyses were conducted to assess the stability of the results [14, 18]. All statistical analyses were carried out via RevMan (Version 5.4.1).

## Results

### Literature search and selection

**Fig. 1:**
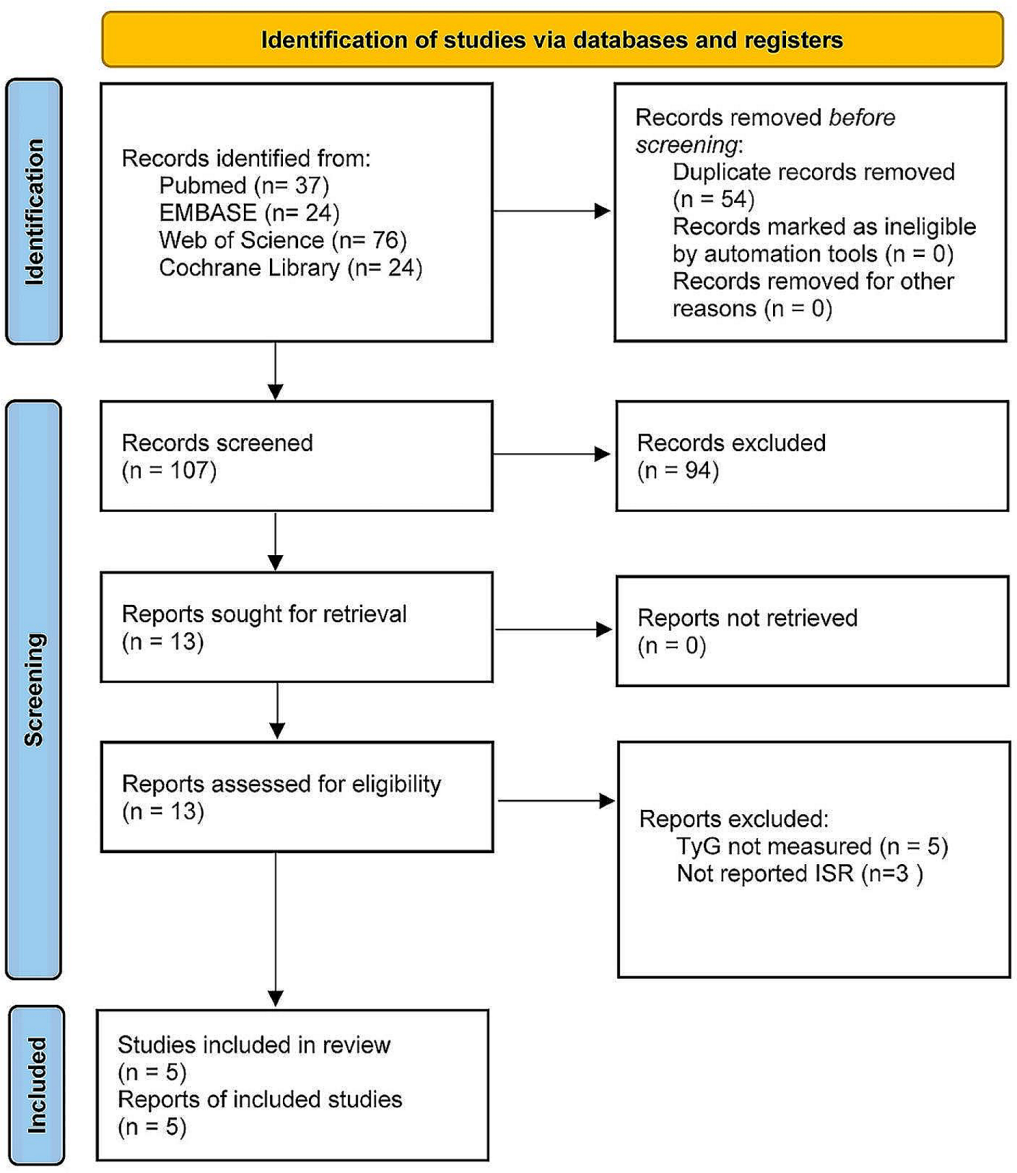
Flow diagram of study selection

Initially, 161 articles were identified. After removing duplicate articles, the number was reduced to 107. Upon reviewing titles and abstracts, 94 articles were excluded for being irrelevant to the research topic. Full-text analysis was performed on the remaining thirteen articles, revealing that five did not report the TyG index, and three did not report ISR outcomes. Ultimately, five studies were included (Fig. 1).

### Study characteristics and quality assessment

Five observational studies, comprising 3,912 participants who had previously undergone stent implantation and had no ISR at baseline, were included. Among these studies, three were conducted in China [10, 19, 20] and two in Turkey [9, 21]. Participants’ ages ranged from 56 to 63 years, with male proportions varying from 60 to 80% and diabetes prevalence between 0% and 50%. All five studies analyzed the TyG index as a continuous variable [9, 10, 19–21], with two additionally treating it as a categorical variable [19, 20]. All the studies conducted adjustments for confounding factors (Tables 1 and 2). The NOS scores for all five studies were above 6, indicating the high quality of the studies. (Details of the NOS scoring can be found in Additional file 1: Table S1 and S2).

**Table 1:** Characteristics of the research protocol

Author	Design	TyG index analysis	Variables adjusted	Outcome OR/HR, 95%CI
Ferik [9]	Case-control study	Continuous	Gensini score, SYNTAX score, WBC count, TC, LDL-c, UA	The TyG index is associated with ISR (OR:1.328,95%CI 1.103–1.654).
Guo [20]	Cohort study	Continuous and categorized	Sex, Ages, BMI, PrePCI, PAD, MVD, hs-CRP, eGFR, lesion length, stent length	The higher TyG index was associated with an increased risk of ISR. (categorical variables: HR: 1.738, 95%CI = 1.250–2.417; continuous variables: HR:1.423, 95%CI 1.159–1.748).
Klay [21]	Cohort study	Continuous	DM, CHD history, type of stent;	Multivariant analysis revealed that TyG index (OR: 4.144, 95%CI = 1.331–12.906) significantly predicted the development of ISR
Yingle Wu [10]	Case-control study	Continuous	Sex, Ages, BMI, HBP, DM, PrePCI, Curremt smoking, HDL, LDL, Lpa, UA, hs-CRP, HCY, Syntax score, Minimal stent diameter	The TyG index was associated with ISR (OR = 1.766, 95% CI = 1.055– 2.957)
Yong Zhu [19]	Case-control study	Continuous and categorized	Sex, Ages, BMI, HBP, DM, PrePCI, LVEF, Hs-CRP, SYNTAX score, target vessel, the application of intracoronary imagine; DES-sirolimus; stent lenght, minimal stent diameter	The higher TyG index was independently associated with an increased risk of DES-ISR. (continuous variables: OR = 1.424, 95%CI 1.16–1.818; categorical variables: OR = 1.634, 95%CI = 1.125–2.374)

Abbreviations: WBC white blood cell, TC total cholesterol, PAD peripheral artery disease, LDL-c low-density lipoprotein cholesterol, UA uric acid, MVD multivessel Disease, BMI body mass index, Pre-PCI previous PCI, hs-CRP high-sensitivity c-reactive protein, eGFR estimated Glomerular Filtration Rate, DM Diabetes Mellitus, Hcy Homocysteine, HDL high-density Lipoprotein, Lpa Lipoprotein(a), CHD coronary heart disease, OR odds ratio, DES drug-eluting stent, HR hazard ratio

**Table 2:** Characteristics of participants

Author	Year	Follow-up	Country	Characteristic of participants	Number of participants	Age(years)		Male(%)		Diabetes(%)	
						ISR	NISR	ISR	NISR	ISR	NISR
Ferik [9]	2022	NR	Turkey	CCS	214	65 ± 9.9	63.6 ± 10.3	69	74.5	0	0
Guo [20]	2023	5years	China	CCS	1414	57.23 ± 8.93	58.19 ± 9.41	77.87	78.7	50	40.71
Klay [21]	2021	6-12months	Turkey	CHD	124	56.6 ± 8.9	57.3 ± 9.1	81.3	61.8	43.8	21.10
Yingle Wu [10]	2022	NR	China	ACS	586	56.66 ± 9.88	57.51 ± 9.95	77	75	59	47
Yongzhu [19]	2021	NR	China	ACS	1574	58.95 ± 9.81	58.29 ± 9.32	77.1	77.4	46.6	32.2

Abbreviations: NR not report, CCS chronic coronary syndrome, ACS acute coronary syndrome, CHD coronary heart disease, ISR in-stent restenosis

### TyG index and ISR in CHD patients

**Fig. 2:**
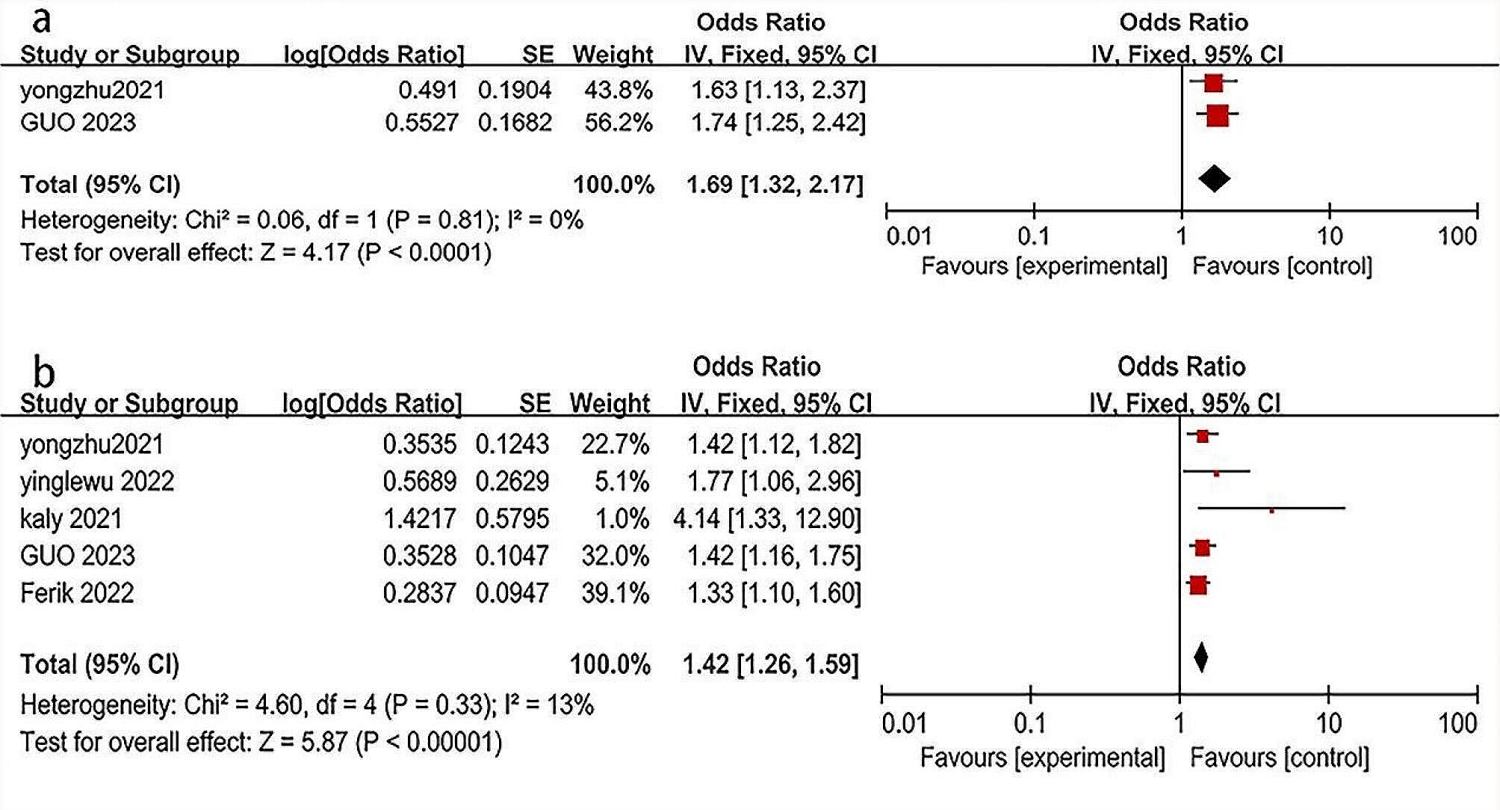
Forest plot of correlation between the TyG index and ISR in CHD patients. (**a**) Analysing the TyG index categorically. (**b**) Treating the TyG index as a continuous variable

Analysis of data from five studies showed that, in the context of CHD patients, each incremental unit in the TyG index, when treated as a continuous variable, corresponded to a 42% elevation in ISR risk (OR 1.42, 95% CI 1.26–1.59, I²=13%, *p* < 0.005, Fig. 2a) [9, 10, 19–21]. Moreover, in studies where the TyG index was categorized, results indicated a significantly higher risk of ISR in CHD patients with the highest TyG index compared to those with the lowest TyG index (OR: 1.69, 95% CI 1.32–2.17, I²=0, Fig. 2b) [19, 20]. Sensitivity analysis confirmed the robustness of these results irrespective of the analytical approach to the TyG index. (For more details on sensitive analysis, refer to Additional file 1: Figure S1).

### TyG index and ISR in CCS and ACS patinents

**Fig. 3:**
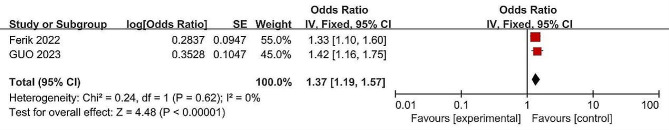
Forest plot of correlation between TyG index and ISR in patients with CCS

**Fig. 4:**
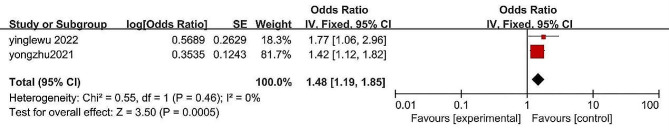
Froest plot of correlation between TyG index and ISR in ACS patients

In the two studies focusing on CCS patients, the combined results showed that for each unit increase in the TyG index, the risk of ISR in patients increased by 37% (95% CI 1.19–1.57, I²=0%, *p* < 0.005, Fig. 3) [9, 20]. A similar trend was observed in studies focusing on ACS patients (OR:1.48, 95% CI 1.19–1.85, I²=0, *p* < 0.005, Fig. 4) [10, 19]. Sensitivity analysis confirmed the reliability of these results (Additional file 1: Figure S2 and S3).

### Publication bias

**Fig. 5:**
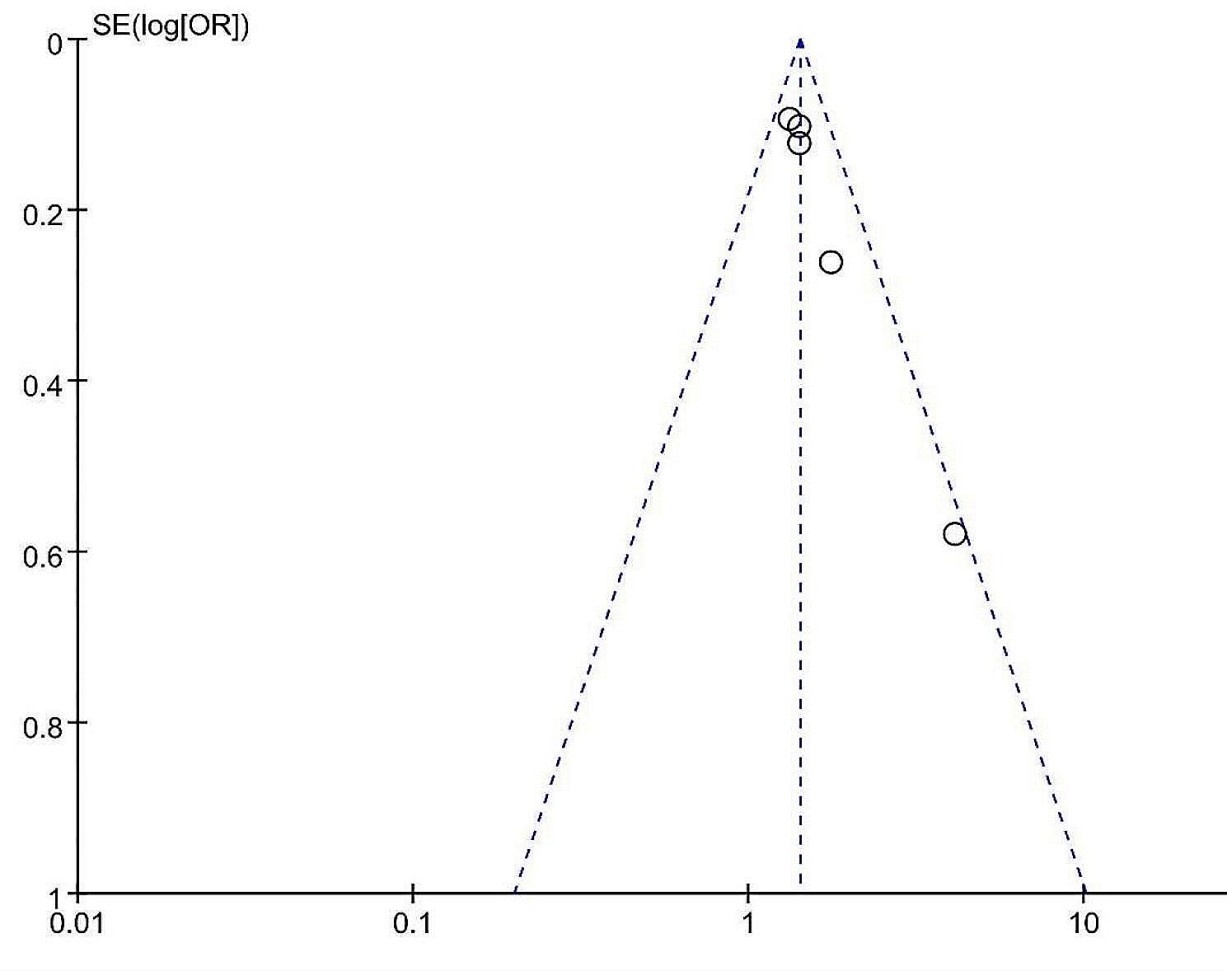
The funnel plot for assessing publication bias when TyG index is a continuous variable

**Fig. 6:**
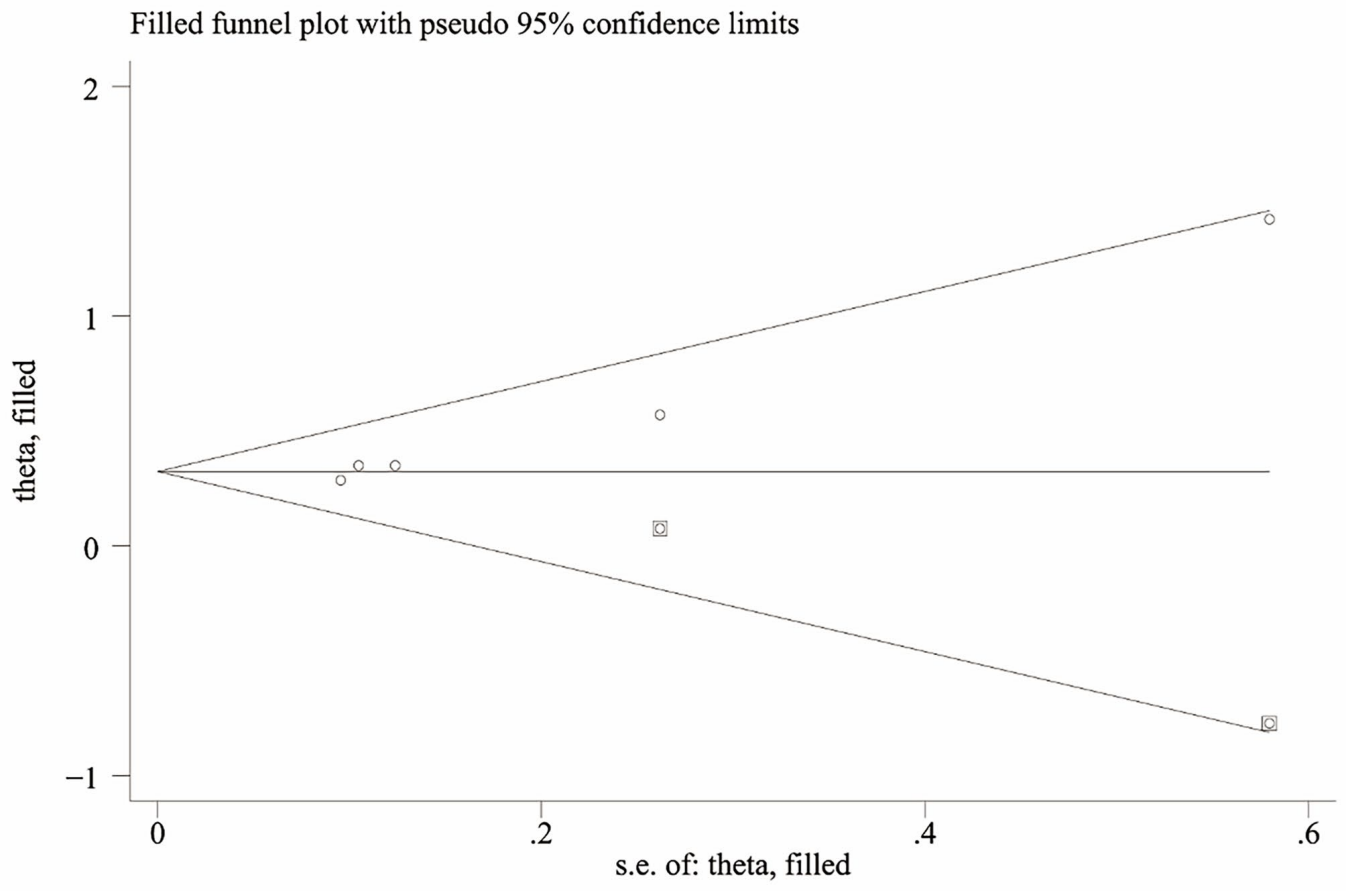
The funnel plot for evaluating publication bias after using trim and fill method

The funnel plot’s visual inspection indicated asymmetry, suggesting the likelihood of significant publication bias (Fig. 5). This was further supported by Egger’s test (*p* = 0.005). The application of the trim-and-fill method added two studies, and the new combined results remained consistent with the previous findings (OR = 1.382, 95% CI 1.234–1.548, *p* = 0.000, Fig. 6). Therefore, this publication bias may not significantly impact the overall results [22].

## Discussion

### Main finding

By integrating the results of five retrieved studies, we have drawn the following conclusions: (1) The TyG index is significantly associated with ISR; (2) Patients with elevated TyG index have a heightened propensity for ISR; (3) Subgroup analysis revealed that this association was unaffected by the type of coronary heart disease. Additionally, the inclusion of five studies may present a certain degree of publication bias. We supposed that small-study effects may be a predominant contributor to publication bias. Nevertheless, it is important to take into account that the asymmetry observed in the funnel plot could also be attributed to the chance, associated with a smaller number of studies.

### The underlying mechanisms

As previously mentioned, the TyG index is related to IR [8]. This led us to reasonably hypothesize that IR may be a contributing factor to the occurrence of ISR. Firstly, the impact of insulin on blood vessels is intricate. Physiologically, it can activate the PI3K pathway to stimulate endothelial cells to produce nitric oxide (NO), which inhibits smooth muscle cell migration, prevents intimal hyperplasia, and reduces platelet adhesion and aggregation. In ISR, insulin downregulates the PI3K/AKT pathway, primarily stimulating the MAPK pathway, resulting in decreased NO release, which in turn promotes smooth muscle proliferation, cellular migration, and plaque formation. Secondly, IR-induced release of pro-inflammatory factors and free fatty acids may also contribute to the pathophysiology of ISR [23, 24].

### TyG index and IR indicators

It must be acknowledged that the correlation between IR and ISR is not a recent discovery. More than 20 years ago, Piatti P and colleagues found that patients with ISR exhibited significant insulin resistance [25]. However, despite the established correlation between IR and ISR, exploring how the TyG index relates to ISR holds value. Firstly, the TyG index computation, based solely on fasting blood glucose and triglyceride concentrations, is a streamlined alternative to HOMA-IR, which necessitates fasting insulin readings, often inaccessible in various healthcare settings. The emergence of the TyG index thus offers an accessible tool for IR assessment, especially in regions with limited healthcare infrastructure. Secondly, its concordance with the HEC demonstrates the reliability in accurately gauging IR [26, 27]. Lastly, the practicality and cost-effectiveness of the TyG index endorse its application for extensive clinical diagnostics, underscoring the ongoing significance of exploring its connection with ISR.

### TyG Index and Cardiovascular Disease

The TyG index extends its applicability beyond the realm of coronary heart disease, playing a significant role in the areas of heart failure and arrhythmias. Researchers have discovered in their studies on the risk factors for atrial fibrillation that patients with this condition tend to have higher TyG index levels. Moreover, a meta-analysis has shown that individuals with heart failure also exhibit elevated TyG index levels compared to healthy individuals [28–30]. Consequently, the TyG index is increasingly recognized as an indispensable tool for assessing cardiovascular risk, demonstrating its efficacy in prognosticating CHD, heart failure, arrhythmias, and associated clinical outcomes. As research progresses, the TyG index is anticipated to ascend as a paramount biomarker in the cardiovascular health landscape, guiding the development of nuanced, patient-centered treatment modalities across the globe.

### Strengths and imitations

Our study pioneers in demonstrating the predictive utility of the TyG index for ISR through a meta-analytical approach. Given the scarcity of related research, our analysis serves to partially fill the knowledge void in this area. Furthermore, our findings provide robust theoretical support for the utilization of the TyG index as a screening tool for ISR in community hospitals and regions with limited medical resources. Ultimately, our research reaffirms the vital importance of the TyG index in the cardiovascular domain. Several limitations of our study must be recognized: firstly, being a meta-analysis predominantly comprising case-control studies, it was unable to conclusively determine causality, thereby weakening the robustness of the evidence. Secondly, the included literature may have some degree of publication bias, potentially affecting the accuracy of the results. Thirdly, the quantity of data from the included studies was somewhat limited, highlighting the need for additional research to confirm our conclusions.

## Conclusions

In conclusion, our research establishes that the TyG index shows a significant association with ISR. This correlation is unaffected by the type of CHD. Moreover, our findings reveal that the TyG index is a helpful instrument to identify patients at increased risk of ISR, especially beneficial in regions where medical resources are sparse. However, further investigations are imperative to validate our findings.

### Electronic supplementary material

Below is the link to the electronic supplementary material.

Supplementary Material 1

## Data Availability

All data generated or analysed during this study are included in this published article [and its supplementary information files].

## Abbreviations

IR: Insulin resistance
PCI: percutaneous coronary intervention
TyG index: triglyceride-glucose index
ISR: In-stent restenosis
CCS: Chronic coronary syndrome
ACS: Acute Coronary Syndrome
PRISMA: Preferred Reporting Items for Systematic Reviews and Meta-analyses
NOS: Newcastle-Ottawa Scale
CI: Confidence interval
OR: odds ratio
RR: relative risk
HR: hazard ratio

## References

[1] Bax JJ, Achenbach S, Valgimigli M, Muneretto C, Masip J, Mahfoud F, Hatala R, Hasdai D, Gilard M, Svitil P (2020). 2019 ESC guidelines for the diagnosis and management of chronic coronary syndromes. Eur Heart J.

[2] Tao S, Tang X, Yu L, Li L, Zhang G, Zhang L, Huang L, Wu J. Prognosis of coronary heart disease after percutaneous coronary intervention: a bibliometric analysis over the period 2004–2022. Eur J Med Res 2023, 28(1). 10.1186/s40001-023-01220-5 PMC10472664 37658418

[3] Alfonso F, Coughlan JC, Giacoppo D, Kastrati A, Byrne RB (2022). Management of in-stent restenosis. EuroIntervention.

[4] Zheng J-F, Guo T-T, Tian Y, Wang Y, Hu X-Y, Chang Y, Qiu H, Dou K-F, Tang Y-D, Yuan J-Q (2020). Clinical characteristics of early and late drug-eluting stent in-stent restenosis and mid-term prognosis after repeated percutaneous coronary intervention. Chin Med J.

[5] Giustino G, Colombo A, Camaj A, Yasumura K, Mehran R, Stone GW, Kini A, Sharma SK (2022). Coronary In-Stent restenosis. J Am Coll Cardiol.

[6] Ormazabal V, Nair S, Elfeky O, Aguayo C, Salomon C, Zuñiga FA. Association between insulin resistance and the development of cardiovascular disease. Cardiovasc Diabetol 2018, 17(1). 10.1186/s12933-018-0762-4 PMC6119242 30170598

[7] Guerrero-Romero F, Simental-Mendía LE, González-Ortiz M, Martínez-Abundis E, Ramos-Zavala MaG, Hernández-González SO, Jacques-Camarena O, Rodríguez-Morán M (2010). The product of triglycerides and glucose, a simple measure of insulin sensitivity. Comparison with the Euglycemic-Hyperinsulinemic Clamp. J Clin Endocrinol Metabolism.

[8] Liang S, Wang C, Zhang J, Liu Z, Bai Y, Chen Z, Huang H, He Y. Triglyceride-glucose index and coronary artery disease: a systematic review and meta-analysis of risk, severity, and prognosis. Cardiovasc Diabetol 2023, 22(1). 10.1186/s12933-023-01906-4 PMC10327356 37415168

[9] Kurmuş Ferik Ö, Yetiş Sayın B, Akbuğa K, Zorlu Ç (2022). Association between insulin resistance estimated by triglyceride glucose index and In-Stent restenosis in non-diabetic patients. E-J Cardiovasc Med.

[10] Wu Y, Du L, Fan M, Chen X, Tang Y, Wang Y, Wang K, Wang S, Li G (2022). Association between oral infections, triglyceride-glucose index, and in‐stent restenosis. Oral Dis.

[11] Page MJ, McKenzie JE, Bossuyt PM, Boutron I, Hoffmann TC, Mulrow CD, Shamseer L, Tetzlaff JM, Akl EA, Brennan SE et al. The PRISMA 2020 statement: an updated guideline for reporting systematic reviews. BMJ 2021. 10.1136/bmj.n71 PMC8005924 33782057

[12] The Newcastle-Ottawa. Scale (NOS) for assessing the quality of nonrandomised studies in meta-analyses [https://www.ohri.ca/programs/clinical_epidemiology/oxford.asp].

[13] Kwok CS, Sherwood MW, Watson SM, Nasir SB, Sperrin M, Nolan J, Kinnaird T, Kiatchoosakun S, Ludman PF, de Belder MA (2015). Blood transfusion after percutaneous coronary intervention and risk of subsequent adverse outcomes. JACC: Cardiovasc Interventions.

[14] Hernandez AV, Marti KM, Roman YM (2020). Meta-analysis. Chest.

[15] Tufanaru C, Munn Z, Stephenson M, Aromataris E (2015). Fixed or random effects meta-analysis? Common methodological issues in systematic reviews of effectiveness. Int J Evid Based Healthc.

[16] Nikolakopoulou A, Mavridis D, Salanti G (2014). How to interpret meta-analysis models: fixed effect and random effects meta-analyses. Evid Based Ment Health.

[17] Jennions MD, MØLler AP (2007). Publication bias in ecology and evolution: an empirical assessment using the ‘trim and fill’ method. Biol Rev.

[18] Rong Y, Chen L, Zhu T, Song Y, Yu M, Shan Z, Sands A, Hu FB, Liu L (2013). Egg consumption and risk of coronary heart disease and stroke: dose-response meta-analysis of prospective cohort studies. BMJ.

[19] Zhu Y, Liu K, Chen M, Liu Y, Gao A, Hu C, Li H, Zhu H, Han H, Zhang J et al. Triglyceride-glucose index is associated with in-stent restenosis in patients with acute coronary syndrome after percutaneous coronary intervention with drug-eluting stents. Cardiovasc Diabetol 2021, 20(1). 10.1186/s12933-021-01332-4 PMC8268452 34238294

[20] Guo X, Shen R, Yan S, Su Y, Ma L (2023). Triglyceride-glucose index for predicting repeat revascularization and in-stent restenosis in patients with chronic coronary syndrome undergoing percutaneous coronary intervention. Cardiovasc Diabetol.

[21] Kalyoncuoglu M, Ozkan A, Kaya A, Yuksel Y, Dogan N, Gurmen A. A new predictor of in-stent restenosis in patients undergoing elective percutaneous coronary intervention: triglyceride glucose İndex. Int J Cardiovasc Acad 2021, 7(2).

[22] Meng Y, Zeng F, Sun H, Li Y, Chen X, Deng G (2022). Clinical characteristics and outcomes of patients with COVID-19 and psoriasis. J Med Virol.

[23] Shoelson SE (2006). Inflammation and insulin resistance. J Clin Invest.

[24] Li H, Meng Y, He S, Tan X, Zhang Y, Zhang X, Wang L, Zheng W. Macrophages, Chronic Inflammation, and Insulin Resistance. *Cells* 2022, 11(19). 10.3390/cells11193001 PMC9562180 36230963

[25] Piatti P, Di Mario C, Monti LD, Fragasso G, Sgura F, Caumo A, Setola E, Lucotti P, Galluccio E, Ronchi C (2003). Association of insulin resistance, hyperleptinemia, and impaired nitric oxide release with in-stent restenosis in patients undergoing coronary stenting. Circulation.

[26] Son DH, Lee HS, Lee YJ, Lee JH, Han JH (2022). Comparison of triglyceride-glucose index and HOMA-IR for predicting prevalence and incidence of metabolic syndrome. Nutr Metab Cardiovasc Dis.

[27] Tahapary DL, Pratisthita LB, Fitri NA, Marcella C, Wafa S, Kurniawan F, Rizka A, Tarigan TJE, Harbuwono DS, Purnamasari D (2022). Challenges in the diagnosis of insulin resistance: focusing on the role of HOMA-IR and Tryglyceride/glucose index. Diabetes Metab Syndr.

[28] Khalaji A, Behnoush AH, Khanmohammadi S, Ghanbari Mardasi K, Sharifkashani S, Sahebkar A, Vinciguerra C, Cannavo A (2023). Triglyceride-glucose index and heart failure: a systematic review and meta-analysis. Cardiovasc Diabetol.

[29] Liu X, Abudukeremu A, Jiang Y, Cao Z, Wu M, Ma J, Sun R, He W, Chen Z, Chen Y (2023). U-shaped association between the triglyceride-glucose index and atrial fibrillation incidence in a general population without known cardiovascular disease. Cardiovasc Diabetol.

[30] Tang Q, Guo XG, Sun Q, Ma J (2022). The pre-ablation triglyceride-glucose index predicts late recurrence of atrial fibrillation after radiofrequency ablation in non-diabetic adults. BMC Cardiovasc Disord.

