# Supplementary material for: The association between the triglyceride-glucose index and in-stent restenosis in patients undergoing percutaneous coronary intervention: a systematic review and meta-analysis
Source: BMC Cardiovasc Disord. 2024 May 3;24:234. doi: 10.1186/s12872-024-03903-1 (PMC11067166; doi:10.1186/s12872-024-03903-1)
Supplement: Supplementary file 1 — Supplementary Material 1 [file 12872_2024_3903_MOESM1_ESM.docx]

**Additional file**

**Search strategies**

***1.Embase***

#1.'triglyceride-glucose index'/exp

#2. 'fasting glucose and triglyceride (tyg index)'/exp OR 'fasting glucose and triglyceride (tyg index)' OR 'fasting glucose and triglycerides (tyg index)' OR 'fasting plasma glucose and triglycerides (tyg index)' OR 'fasting plasma glucose and triglycerides index' OR 'fasting triglyceride-glucose index' OR 'fasting triglycerides-glucose (tyg) index' OR 'fasting triglycerides-glucose index' OR 'glucose and triglycerides (tyg index)' OR 'glucose and triglycerides index' OR 'glucose-triglyceride index (tyg)' OR 'glucose-triglycerides index' OR 'triglyceride and glucose index' OR 'triglyceride x glucose (tyg) index' OR 'triglyceride-glucose (tyg) index' OR 'triglyceride-glucose index (tyg index)' OR 'triglycerides x glucose (tyg index)' OR 'triglycerides-glucose (tyg) index' OR 'triglycerides-glucose index' OR 'triglycerides-glucose index (tyg index)' OR 'triglycerides-glucose index (tyg)' OR 'tyg index' OR 'triglyceride-glucose index'

#3. 'stent'/exp

#4. 'stent'/exp OR stent OR stents OR stenting

#5.#1 OR #2

#6. #3 OR #4

#7. 5 AND #6

***2.Web of Science***

#1.fasting glucose and triglyceride (Topic) or fasting glucose and triglycerides (Topic) or fasting plasma glucose and triglycerides (Topic) or fasting plasma glucose and triglycerides index (Topic) or fasting triglyceride-glucose index (Topic) or fasting triglycerides-glucose index (Topic) or glucose and triglycerides (Topic) or glucose and triglycerides index (Topic) or glucose-triglyceride index (Topic) or glucose-triglycerides index (Topic) or triglyceride and glucose index (Topic) or triglyceride and glucose index (Topic) or triglyceride x glucose index (Topic) or triglyceride-glucose index (Topic) or triglyceride-glucose index (Topic) or triglycerides x glucose (Topic) or triglycerides-glucose index (Topic) or triglycerides-glucose index (Topic) or triglycerides-glucose index (Topic) or triglycerides-glucose index (Topic) or  TyG index (Topic) or triglyceride-glucose index (Topic)

#2.stent (Topic) or stents (Topic) or stenting (Topic)

#3.#1 AND #2

***3. Pubmend:***

 (((((((((((((((((((((((fasting glucose and triglyceride) OR (fasting glucose and triglycerides)) OR (fasting plasma glucose and triglycerides)) OR (fasting plasma glucose and triglycerides index)) OR (fasting triglyceride-glucose index)) OR (fasting triglycerides-glucose index)) OR (fasting triglycerides-glucose index)) OR (glucose and triglycerides)) OR (glucose and triglycerides index)) OR (glucose-triglyceride index)) OR (glucose-triglycerides index)) OR (triglyceride and glucose index)) OR (triglyceride and glucose index)) OR (triglyceride x glucose index)) OR (triglyceride-glucose index)) OR (triglyceride-glucose index)) OR (triglycerides x glucose)) OR (triglycerides-glucose index)) OR (triglycerides-glucose index)) OR (triglycerides-glucose index)) OR (triglycerides-glucose index)) OR (TyG index)) OR (triglyceride-glucose index)) AND ("Stents"[Mesh] OR stent OR stents OR stenting)

***4, Cochrane Library***

#1 MeSH descriptor: [Stents] explode all trees 5659

#2 (stents):ti,ab,kw OR (stent):ti,ab,kw OR (stenting):ti,ab,kw (Word variations have been searched) 18598

#3 Any MeSH descriptor in all MeSH products

#4 (fasting glucose and triglyceride):ti,ab,kw OR (fasting glucose and triglycerides):ti,ab,kw OR (fasting plasma glucose and triglycerides):ti,ab,kw OR (fasting plasma glucose and triglycerides index):ti,ab,kw OR (fasting triglyceride-glucose index):ti,ab,kw (Word variations have been searched) 6506

#5 (fasting triglycerides-glucose index):ti,ab,kw OR (glucose and triglycerides):ti,ab,kw OR (glucose and triglycerides index):ti,ab,kw OR (glucose-triglyceride index):ti,ab,kw OR (triglyceride and glucose index):ti,ab,kw (Word variations have been searched) 11290

#6 (triglyceride x glucose index):ti,ab,kw OR (triglyceride-glucose index):ti,ab,kw OR (triglycerides x glucose):ti,ab,kw OR (triglycerides-glucose index):ti,ab,kw OR (TyG index):ti,ab,kw (Word variations have been searched) 1520

#7 (triglyceride-glucose index):ti,ab,kw (Word variations have been searched) 126

#8 #4 OR #5 OR #6 OR #7 11298

#9 #1 OR #2 18598

#10 #8 AND #9 24

**NEWCASTLE - OTTAWA QUALITY ASSESSMENT SCALE**

**COHORT STUDIES**

**Selection**

**1.Representativeness of the exposed cohort;**

**2.Selection of the non exposed cohort;**

**3.Ascertainment of exposure;**

**4.Demonstration that outcome of interest was not present at start of study;**

**Comparability**

**1.Comparability of cohorts on the basis of the design or analysis;**

**Outcome**

**1.Assessment of outcome;**

**2.Was follow-up long enough for outcomes to occur;**

**3.Adequacy of follow up of cohort**

**NEWCASTLE - OTTAWA QUALITY ASSESSMENT SCALE**

**CASE CONTROL STUDIES**

**Selection**

**1.Is the case definition adequate?**

**2.Representativeness of the cases**

**3.Selection of Controls**

**4.Definition of Controls**

**Comparability**

**1.Comparability of cases and controls on the basis of the design or analysis**

**Exposure**

**1.Ascertainment of exposure**

**2.Same method of ascertainment for cases and controls**

**3.Non-Response rate**

**Table S 1 NOS of the cohort study**

| **Study** | **Selection** | | | | **Comparability** | **Outcome** | | | **Overall** |
| --- | --- | --- | --- | --- | --- | --- | --- | --- | --- |
|  | **1** | **2** | **3** | **4** | **1** | **1** | **2** | **3** |  |
| **Guo2023** | 1 | 1 | 1 | 0 | 2 | 1 | 1 | 0 | 7 |
| **kaly2021** | 1 | 1 | 1 | 0 | 1 | 1 | 0 | 1 | 6 |

**Table S 2 NOS of the case-control studies**

| **Study** | **Selection** | | | | **Comparability** | **Exposure** | | | **Overall** |
| --- | --- | --- | --- | --- | --- | --- | --- | --- | --- |
|  | **1** | **2** | **3** | **4** | **1** | **1** | **2** | **3** |  |
| **Ferik2022** | 1 | 0 | 1 | 1 | 1 | 1 | 1 | 0 | 6 |
| **yinglewu2022** | 1 | 0 | 1 | 0 | 1 | 1 | 1 | 1 | 6 |
| **yongzhu2021** | 1 | 0 | 1 | 0 | 2 | 1 | 1 | 1 | 6 |


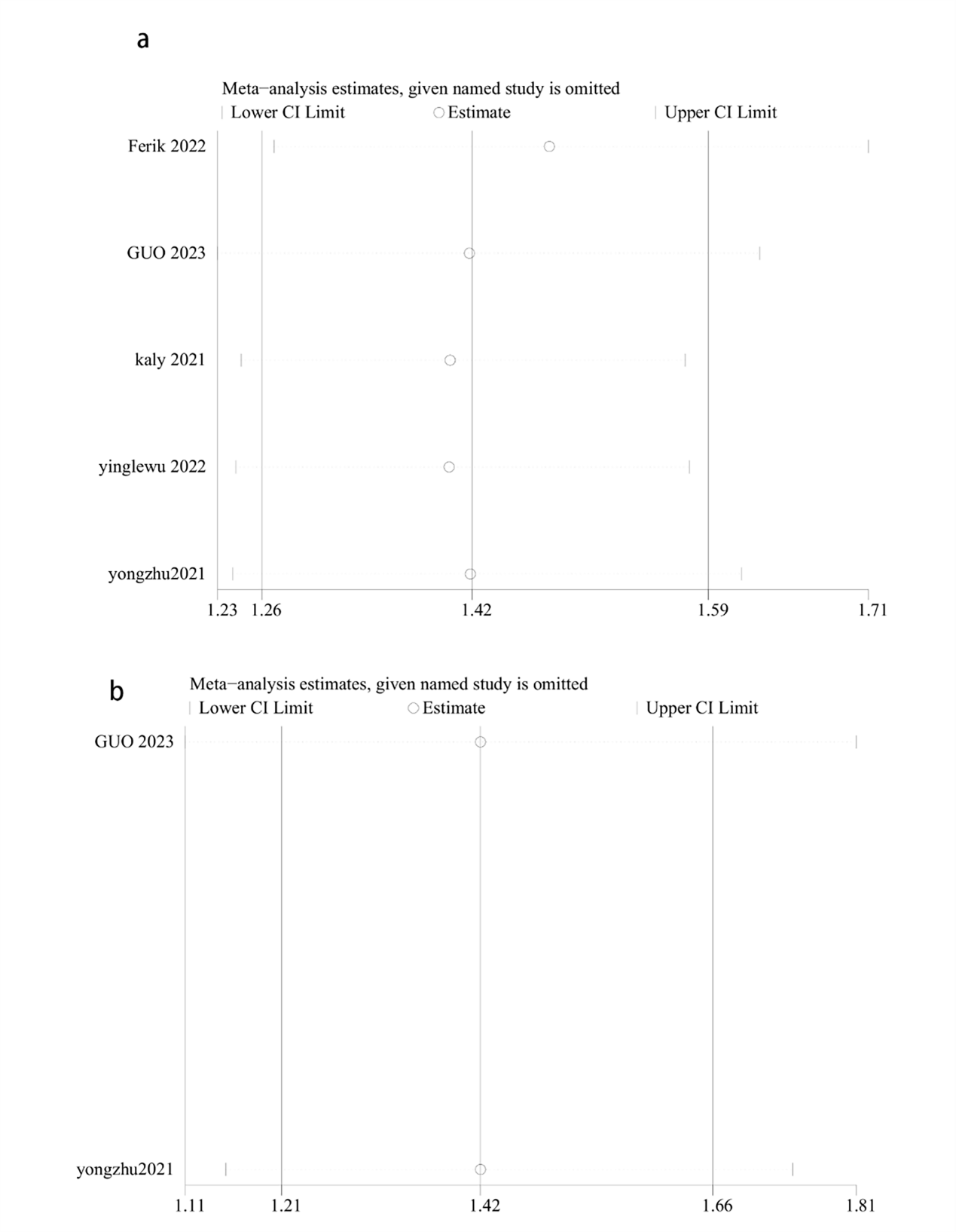


**Figure S 1** Sensitivity analysis of the association between TyG index and ISR restenosis in patients with CHD. a. the TyG index as a continuous variable. b. he TyG index as a categorized variable.


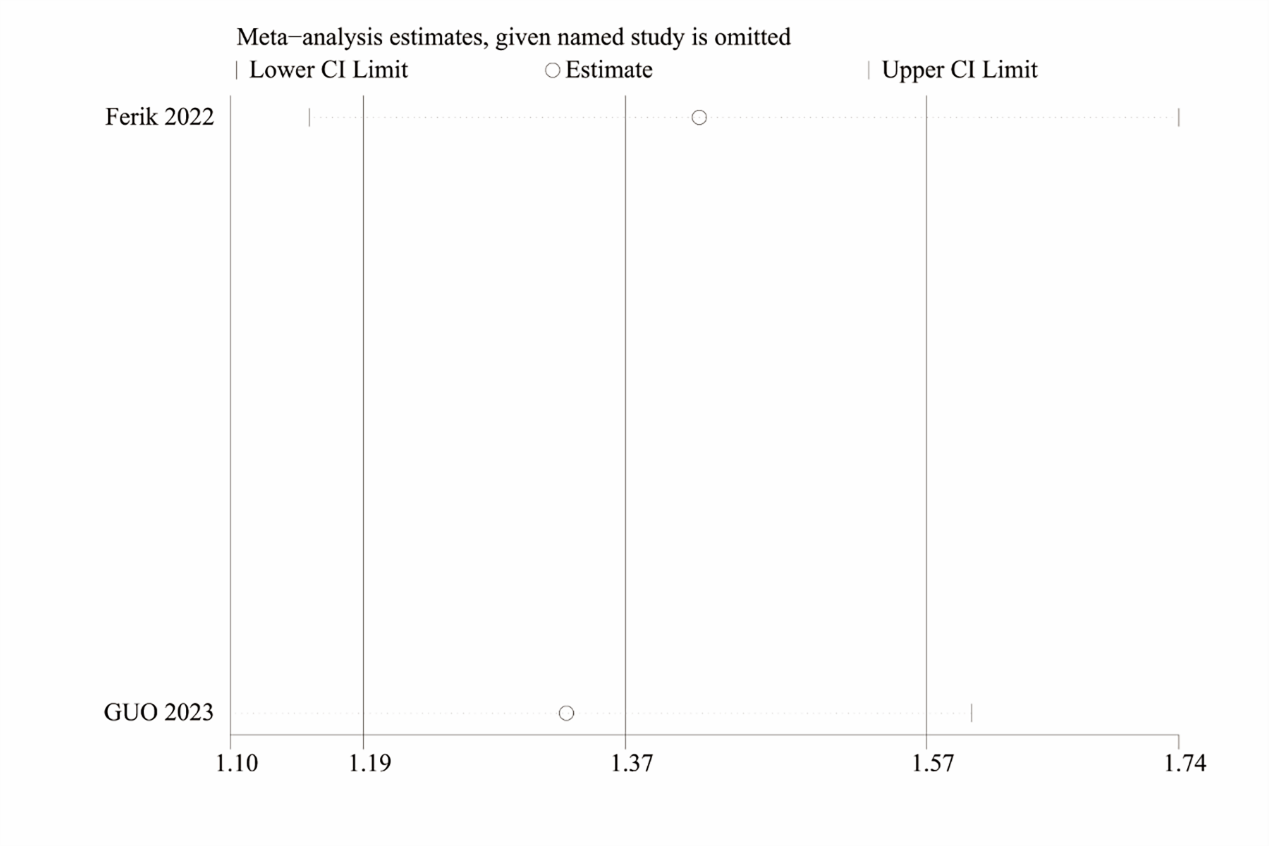


**Figure S 2** Sensitivity analysis of the association between TyG index and ISR restenosis in patients with CCS..


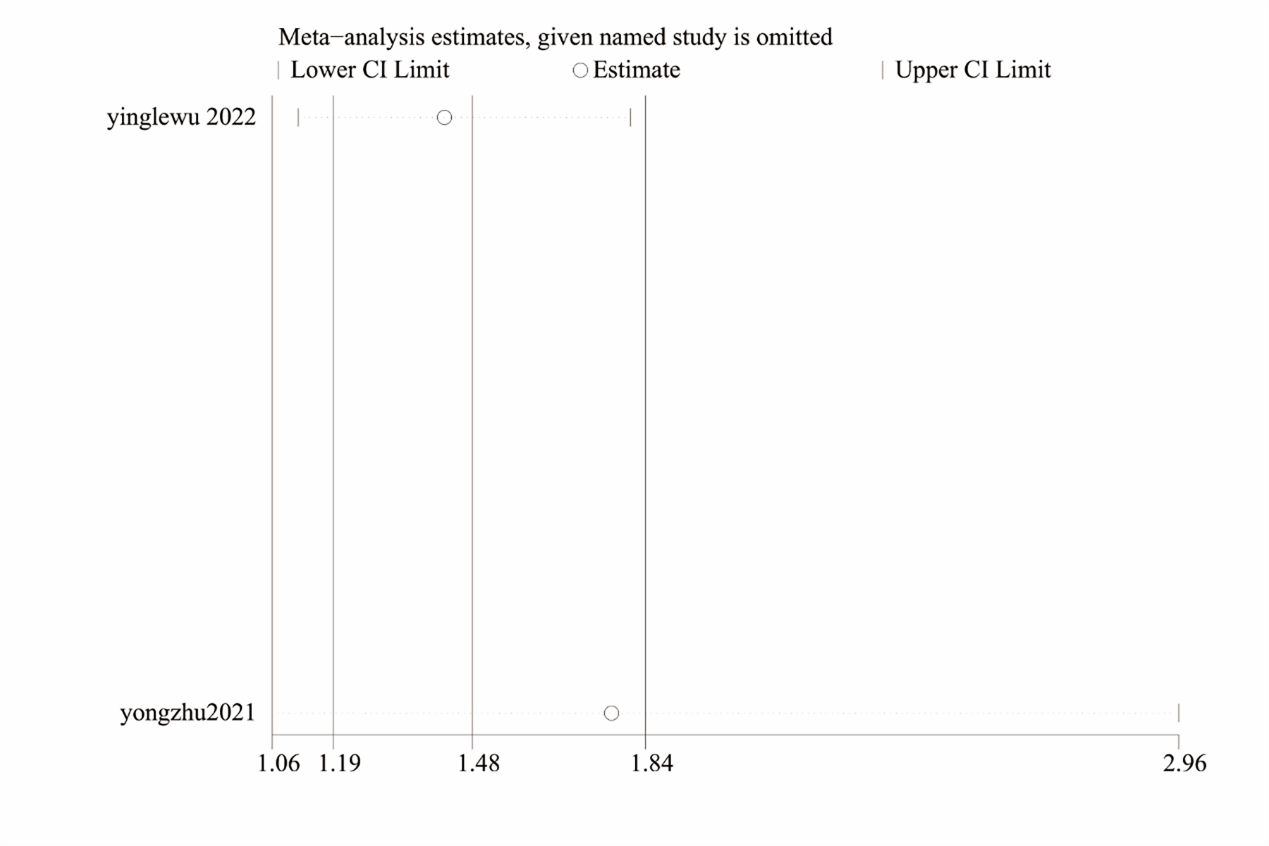


**Figure S 3** Sensitivity analysis of the association between TyG index and ISR restenosis in patients with ACS.
